# English as a foreign language learners’ strategy awareness across proficiency levels from the perspective of self-regulated learning metafactors

**DOI:** 10.3389/fpsyg.2022.1019561

**Published:** 2022-10-10

**Authors:** Anita Habók, Andrea Magyar, Gyöngyvér Molnár

**Affiliations:** ^1^Institute of Education, University of Szeged, Szeged, Hungary; ^2^MTA–SZTE Digital Learning Technologies Research Group, Szeged, Hungary

**Keywords:** EFL learners, strategy awareness, attitude, proficiency level, metafactors, self-regulated learning, metacognition, learning strategies

## Abstract

The previous three decades have seen a growing body of research into language learners’ self-regulated learning (SRL), language learning strategy (LLS) use, and their possible effects on proficiency. This study thus provides insights into the relationship between elementary and low intermediate learners’ perceptions of their self-regulated strategy use in English as a foreign language (EFL) and their attitude to English in relation to their proficiency level. Nine hundred and sixty-six higher proficiency students and 399 lower proficiency students in Year 8 participated in the research. A revised Self-Regulated Foreign Language Learning Strategy Questionnaire (SRFLLSQ), a version of Oxford’s Strategy Inventory for Language Learning (SILL), was completed by the participants. Our findings shed light on higher proficiency learners’ significantly higher level of strategy use. Learners at higher levels relied more strongly on their metacognitive strategies, such as planning, organizing and monitoring their cognitive processes. Our analysis also showed a high correlation between the different factors of metastrategy use (metacognitive, meta-affective, metasocial, and metamotivational) and cognitive, affective, social, and motivational strategy use in relation to attitude and proficiency. A path analysis also reinforced our assumption that metafactors significantly determine learners’ proficiency across strategy use and attitude in both higher and lower-level students. The positive contribution of the metastrategies on their corresponding regulated strategy fields appeared to be robust, thus underlining recent LLS research that emphasizes the role of metafactors in the language learning process.

## Introduction

In recent years, interest in language learning has grown significantly in Hungary. An increasing number of students participate in international projects, travel abroad and have contact with foreign peers, with foreign language (FL) skills having become essential for most jobs. According to Hungary’s National Curriculum, Hungarian students start FL learning at the age of nine and continue for 5 to 9 years in school. However, during the COVID-19 pandemic, most children worldwide were forced to use digital tools for distance learning. Taking part in these classes effectively requires more student autonomy, motivation, self-awareness and self-regulation.

Therefore, self-regulated learning (SRL) has recently gained renewed attention in educational research (Panadero et al., 2015; Panadero, 2017; Schunk and Greene, 2018; Lin et al., 2021; Teng, 2022). From the perspective of educational psychology, effective self-regulated learners are individually capable of activating, organizing, managing and self-monitoring their cognitive processes and systematically directing their learning towards their personal goals (Zimmerman, 2008). Further, there is a growing recognition of the important role that SRL plays in second/foreign language (SL/FL) teaching and learning as teachers encourage students to be self-regulating, independent and goal-oriented and supply them with all the necessary metacognitive, motivational and strategic tools.

In recognition of this, there have been new developments in the last few years in the use of SRL in SL/FL learning and teaching. Oxford (2017) revised language learning strategy (LLS) taxonomy emphasized the self-regulatory features of LLSs and stressed the role of metastrategies that regulate, manage, control and evaluate the language learning process and foster learners’ educational needs in various contexts and settings. Based on her taxonomy, recent research has often investigated LLS use in certain contexts. The strong connection between LLS and academic performance has been reinforced by most of the studies (Ardasheva, 2016; Lee and Heinz, 2016; Bessai, 2018; Teng and Zhang, 2018); however, only a few of them have focused on LLS use from metafactor-oriented perspectives. Many scholars have highlighted that self-regulated, strategic learning can lead to higher academic levels in a number of areas (Ardasheva, 2016; Zhang and Zhang, 2019; Teng and Zhang, 2020). In this study, we thus offer a more complex view of the interrelations and effects among the learning-related variables. Specifically, we involved cognitive, affective, social and motivational strategy factors, and investigated their relationship with proficiency in English as a foreign language (EFL) through attitude to English. In the research, proficiency was indicated by the school marks in the sample. As the central role of attitude has been highlighted in recent studies (Habók and Magyar, 2018a), we also integrated this variable into our investigation.

The aim of the study is therefore to explore the structural relationships of such constructs as SRL strategies and proficiency in the domain of FL learning. To obtain a more comprehensive picture of the individual differences, we investigated the effectiveness of strategy use among students at higher and lower levels of proficiency. Ultimately, we sought answers to four questions. The four aims of the paper are (1) to study which self-regulated LLSs are preferred among the subsamples, (2) to identify any potential differences in the students’ attitudes, (3) to analyze how effectively does metastrategy use influence the corresponding SRL strategies and (4) to discover how strategy use influences English proficiency.

Our research can serve to reinforce the notion that students can be more effective and successful language learners with LLS use. Our study highlights that the significance of that self-regulation process is also an important factor in the learning process. Students can improve their proficiency by using different LLSs. The research design provides separate models for two levels of language learners, who benefit from self-regulated strategy use. Our investigation also stresses the outstanding mediating role of attitude to English, which can likewise have a great effect on proficiency.

## Theoretical background

### The role of self-regulation in the learning process

#### The concept of self-regulation

The notion of self-regulation is not new; it originated from educational psychology in the 1980s. Pintrich (1995) was among the first scholars to define “SRL” as a proactive and goal-directed process, in which learners generate their own learning aims, then manage, organize, control, monitor and supervise their actions to achieve their goals. In recent years, self-regulation of learning has again become a focus of educational research, and there have been immense new refinements and improvements in theorization and model development. Schunk and Greene (2018) summarized the concept from historical and contemporary perspectives and concluded that self-regulation represents “the ways that learners systematically activate and sustain their cognitions, motivations, behaviors and affects toward the attainment of their goals” (p. 1). This definition is consistent with new interpretations of the concept, as it implies that self-regulation involves cognitive, metacognitive, motivational, behavioral and affective aspects. While learners engage with a task, they use various cognitive operations to make sense of the information. Winne (2001) listed the following set of basic cognitive operations: searching, monitoring, assembling, rehearsing and translating (SMART). Beyond cognitive processes, students also use their metacognitive knowledge, regulation and experiences (Efklides, 2018; Zhang and Zhang, 2019). Furthermore, the most important feature of SRL is setting a goal, which directs learners’ activities towards achieving it. In an educational setting, goals can be an improvement or acquisition of new competencies or skills. Another important characteristic is that self-regulation can be regarded as a dynamic and cyclical flow of actions to achieve a desired result; this flow of actions involves feedback loops, through which students can monitor the effectiveness of their learning process (Zimmerman and Schunk, 2011; Schunk and Greene, 2018). Self-regulated learners are thus capable of determining personal goals. They can then metacognitively monitor their cognitive processes while they complete them. They are able to interpret their achievement and modify their study strategies accordingly. After attaining their goals, they can set new goals. In this process, the role of motivation is extremely important, as it regulates whether the learner achieves or abandons his/her goal (Schunk and Zimmerman, 2008; Schunk and Greene, 2018). Hadwin et al. (2011) emphasized that self-regulation is embedded in social situations and involves dynamic interactions between learners and the task, as well as with other peers. Finally, affective and emotional components also play a significant role in managing the self-regulation process (Efklides, 2011; Efklides et al., 2018). Efklides (2018) highlights the affective loop, which both controls and monitors the emotional and affective experiences of the learning process. A positive affective experience inspires the learner to further engage with the learning situation, thus resulting in a more positive SRL cycle.

#### Models of self-regulation

Various models have been developed in recent years with various emphases on these components. Panadero (2017) provided a comprehensive overview of the six most widely acknowledged models: Zimmerman’s Cyclical Phases Model, grounded in socio-cognitive theory (Zimmerman, 2000); Boekaerts’ Adaptable Learning Model and Dual Processing self-regulation model (Boekaerts, 2011); Pintrich’s SRL Model (Pintrich, 2000); two strongly metacognitive-based models, the Winne–Hadwin model (Winne and Hadwin, 1998, 2008; Perry and Winne, 2006) and Efklides’ Metacognitive and Affective Model of Self-Regulated Learning (MASRL; 2011); and Hadwin, Järvelä and Miller’s Socially Shared Regulation of Learning (SSRL; 2011). Most of these models incorporate three main phases: a preparatory phase, which involves: (1) task analysis, planning and goal activation; (2) a performance phase, in which the activity is achieved while the process is self-monitored and self-controlled; and, finally, (3) an appraisal phase, in which the learner reflects on and interprets his/her performance. There are differences in how deeply these phases are articulated. For example, while Zimmerman’s and Pintrich’s models apply different features in the self-regulated phases, Boekaerts and Efklides do not differentiate clearly between the phases and the processes; they interpret self-regulation as an “open” process with recursive phases. All these models include metacognitive, motivational and emotional dimensions of learning and cover further variables, such as learners’ belief, self-efficacy and self-efficiency. The cognitive-metacognitive perspectives are most clearly articulated in the Winne–Hadwin model, the SSRL model and the MASRL model. In contrast, the Boekaerts, Pintrich, and Zimmerman models place emphasis on the role of motivation in SRL. Boekaerts’ model in particular stresses the role of affective components in the SRL process more clearly and discusses how different emotions generate two possible divergent routes as well as various kinds of strategy use (Boekaerts, 2011).

### A taxonomy of self-regulated language learning strategies

In recognition of the importance of self-regulation in the learning processes, there have also been recent changes and developments in language teaching. As regards the strategic aspects of language learning, Rebecca Oxford developed one of the most comprehensive LLS taxonomies (Strategy Inventory for Language Learning; SILL). Her concept sparked a great controversy among scholars (Dörnyei, 2005; Tseng et al., 2006; Griffiths, 2017a,b, 2020; Thomas and Rose, 2018; Thomas et al., 2021a,b) on such issues as definition fuzziness, contentious taxonomies and psychometric properties of the assessment instrument. In the next section, we will discuss the issues that have emerged more comprehensively.

#### Conceptual issues in language learning strategies

The conceptualization of LLSs dates back to the 1980s, when Weinstein and Mayer (1986, p. 315) specified the notion of learning strategies as “behaviors and thoughts that a learner engages in during learning that are intended to influence the learner’s encoding process.” Later, in 1990, Oxford defined them as “specific actions taken by the learner to make learning easier, faster, more enjoyable, more self-directed and more transferable to new situations” (p. 8). In 2015, Griffiths regarded them as “actions chosen by the learners (either deliberately or automatically) for the purpose of learning or regulating the learning of language” (p. 426). These definitions illustrate the dispute over whether learning strategies can be regarded as behavior, thoughts or actions. Dörnyei (2005) highlighted this controversy and argued that a phenomenon cannot be considered as both behavioral and cognitive, so the conceptualization requires a broadened perspective and the entire concept should be replaced with that of self-regulation. This dilemma set off a great debate among scholars. For example, Tseng et al. (2006) proposed that.

the most important aspect of strategic learning is not the exact nature of the specific techniques that students employ, but rather the fact that they choose to exert creative effort in trying to improve their own learning…. [T] he essential aspect of empowering learners is to set into motion the self-regulatory process rather than to offer the instruction of a set of strategies. (p. 95).

Later, Oxford (2017) collected a list of 33 recent definitions, conducted a content analysis and proposed a complex definition of LLSs, which included the self-regulatory nature of the concept:

L2 learning strategies are complex, dynamic thoughts and actions, selected and used by learners with some degree of consciousness in specific contexts in order to regulate multiple aspects of themselves (such as cognitive, emotional and social) for the purpose of (a) accomplishing language tasks; (b) improving language performance or use; and/or (c) enhancing long-term proficiency… (Oxford, 2017, p. 48).

#### A taxonomy of language learning strategies

A taxonomy of LLSs has also been a controversial issue. In line with her development of an original taxonomy, Oxford (1990) worked out an assessment tool, the Strategy Inventory for Language Learning (SILL), which contained her original taxonomy of six strategy constructs: memory, cognitive, metacognitive, compensation, affective and social strategies. SILL focused on specific strategic behaviors for the six fields, and the scale descriptors indicated the frequencies of strategy use (ranging between ‘never’ and ‘always’). Dörnyei (2005) and other scholars (Tseng et al., 2006) later criticized these items as being behavioral, arguing that strategy use cannot be evaluated with the frequency of strategy use, yet it is frequent use of a number of different strategies that produces a high score on SILL. They also pointed out that it is not quantity that matters; instead, it is the quality of strategy use that is essential. As an extreme example, experienced learners often employ only some of the strategies, but in an effective way. Furthermore, inexperienced students use a number of strategies, but ineffectively and rather randomly.

In response to the criticisms and experiences of her taxonomy, Oxford (2017) recently reconsidered and restructured her model based on self-regulation theories. Four main fields of strategies were identified in her Strategic Self-Regulation (S^2^R) Model: cognitive, affective, social and motivational, each of them regulated by the corresponding “master category of metastrategies.” These metastrategies include metacognitive, meta-affective, metasocial and metamotivational strategies. These metastrategies are identical across the four strategy constructs; they comprise (a) paying attention, (b) planning, (c) organizing learning and obtaining resources, and (d) monitoring and evaluating the corresponding sets of strategies. They supervise and regulate the language learning process and foster the language learner’s needs in various contexts and settings (Oxford, 2017). Her reconsidered taxonomy has sparked numerous debates, which some researchers (e.g., Thomas et al., 2022) have interpreted as the start of a ‘third wave’ in strategy research, including previously unexplored theoretical views, challenges in strategy categorization and social influences on strategy use.

#### Research on self-regulated language learning strategies

With the revision of the concept of language learning strategies, strategy research has again come into the light of research and a rich body of literature focused again on the measurement of self-regulated language strategies. Rose et al. (2018) highlight the following three main directions of recent research: (1) self-regulation-oriented strategy research (e.g., Tseng et al., 2006); (2) LLS-oriented strategy investigations (e.g., Ardasheva and Tretter, 2013; Bessai, 2018); and (3) finding new paths for self-regulated learner strategies (e.g., Teng and Zhang, 2016; Habók and Magyar, 2018b).

As the focus of this paper is to investigate EFL strategy awareness from the perspective of SRL across proficiency levels, here we concentrate on the presentation of research that moved towards the area of self-regulation *via* new instrument development or investigated the links between strategic learning and proficiency.

##### Instrument development for regulated LLSs

First, Tseng et al.’s (2006) development of their Self-Regulating Capacity in Vocabulary Learning scale (SRCvoc) demonstrated the shift in research focus from measuring strategy use to exploring underlying processes. The items on their scale show the overall tendencies of learners rather than their specific strategy use. The unidimensional construct of the scale suggests that the concept of self-regulation from educational psychology can be effectively transferred to FL learning.

Ardasheva and Tretter (2013) modified and validated Oxford (1990) SILL for younger EFL learners by developing a shorter version of 28 items named the SILL-ELL Student Form. The importance of this study lies in its precise demonstration of how SILL can be implemented and validated to form a more robust assessment tool for diverse EFL contexts.

Teng and Zhang (2016) also created and validated a new instrument named Writing Strategies for Self-Regulated Learning Questionnaire (WSSRLQ). They analyzed EFL learners’ self-reported use of metacognitive, cognitive and social behavior strategies in the learning to write environment. They found that both metacognitive and cognitive strategies directly affected the writing achievement of the learners under examination. They also discovered that motivational regulation showed a weak but direct effect on it, while social behavioral strategies did not reinforce it (Teng and Zhang, 2018; Teng, 2022).

Habók and Magyar also developed and validated a measurement tool based on Oxford’s S^2^R Model (Self-Regulated Foreign Language Learning Strategy Questionnaire; SRFLLSQ; Habók and Magyar, 2018b). A construct of a five-factor model with metacognitive, cognitive, meta-affective, meta-sociocultural-interactive and sociocultural-interactive factors was validated. As the affective construct did not fit into the data, modification recently became necessary to align the measurement tool with Oxford’s revised theory (Oxford, 2017; Habók et al., 2022). The final results showed the complex nature of the LLS construct, which involves cognitive, affective, social and motivational factors, each regulated by their corresponding metastrategy.

##### Research on the relationship between foreign language learning strategies and proficiency

The other line of recent research has been to explore the relationship between strategic learning and proficiency. A strong link between LLS and academic performance has been reinforced by most of the studies (Bessai, 2018; Teng and Zhang, 2018, 2020; Taheri et al., 2019). Bessai (2018) found that more proficient students employed various strategy types compared to less proficient learners. The study also pointed out the strong correlation between strategy use and LLS use. Taheri et al. (2019) reported on a significant correlation between cognitive, social and compensation strategy use and achievement in English as a second language (ESL). Teng and Zhang (2018) reinforced the significant correlation between writing proficiency and cognitive, metacognitive and motivational regulation strategies. Teng and Zhang (2020) also noted that writing proficiency had a significant impact on learners’ test results.

Studies have also investigated how less and more proficient learners differ in their metacognitive awareness of themselves as learners and in their strategy use in language learning (Lee and Heinz, 2016; Bessai, 2018). While less proficient students reported using more compensation strategies, more proficient learners employed metacognitive strategies more frequently (Bessai, 2018). Lee and Heinz (2016) also found more extensive use of metacognitive strategies in the more proficient group. Chen and Aziz (2021) likewise confirmed metacognitive strategy preference among high-achieving EFL learners. Sánchez (2019) reported the greatest use of social and metacognitive strategies among both less and more proficient students.

Not many studies have applied advanced modelling techniques to explore a more comprehensive view of the interrelations and effects among the strategy use constructs and FL proficiency. Ardasheva (2016) explored the interrelations between LLSs and English proficiency and found that only metacognitive strategies indicated a significant direct effect on academic English proficiency (β = 0.09, *p* < 0.05). The findings also indicated a negative impact of cognitive strategies on English proficiency. As regards the effects of other strategies, they were also negative, although statistically significant.

## Objectives of the study

**Figure 1:**
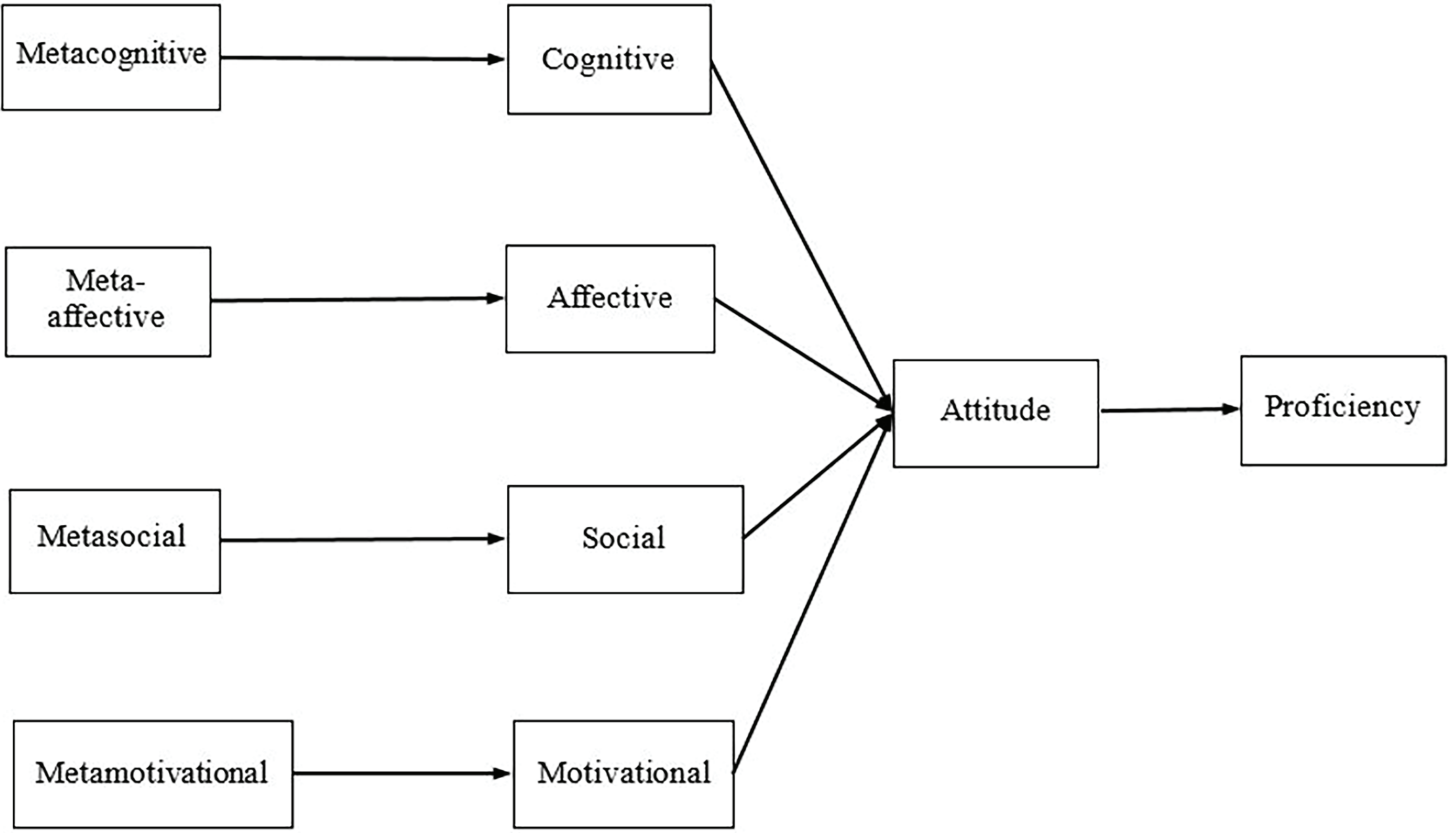
The theoretical model of strategy effects on proficiency through attitude.

The main objective of the study was to analyze the connections and effects between elementary and low intermediate students’ perceptions of their EFL strategy use and their attitude to English in relation to their proficiency levels. A theoretical model was developed (Figure 1) based on Oxford (2017) taxonomy, which involved four exogenous factors (metacognitive, meta-affective, metasocial and metamotivational strategies) and six endogenous constructs (cognitive, affective, social and motivational strategy uses, attitude to English, and proficiency in English, which was indicated by EFL school marks). In this hierarchical model, we proposed that cognitive, affective, social and motivational metastrategies have an indirect effect on proficiency, mediated by the corresponding self-regulated LLS and attitude. The central role of attitude has been demonstrated in previous studies, thus justifying its integration into this model again (Habók and Magyar, 2018a; Habók et al., 2022).

To obtain a more comprehensive view, we investigated the model among elementary and low intermediate learners. Using model analysis, we were seeking the answer to the following research questions:

1. Which self-regulated LLSs are most employed among the two subsamples?
2. Is there a significant difference in attitude to EFL learning between the two subsamples?
3. How effectively does the use of metastrategies affect the corresponding self-regulated LLSs in the groups of students under examination?
4. Does SRL strategy use affect students’ English proficiency?

## Materials and methods

### Participants

A total of 1,365 lower secondary school students in 66 schools were involved in the research. All participants were Year 8 students (14-year-olds). Year 8 students are in their final year of lower secondary education. From Year 9, they move on to upper secondary school, which is a new school level with different school buildings, classmates and teachers. Another reason why 14-year-old students participated in the study is that there are considerable differences at this age between the low-and high-ability students and their strategy use might be very different. We thus managed to demonstrate a more diverse range with this sample. In our study, students were divided into two groups based on their proficiency. Proficiency was indicated by their EFL school mark. School marks in Year 8 are of particular importance because they form the basis for admission to upper secondary education. As recent studies have used various indicators, such as self-rating, achievement test results and school mark, this form is a valid indicator (Habók and Magyar, 2018a; Sánchez, 2019; Bećirović et al., 2021). Thus, the less proficient students (the elementary EFL learners) had a satisfactory or average school mark (2 or 3 on a scale of 1 to 5, with 5 as the top mark) (*N* = 399), while the more proficient students (the low intermediate EFL learners) had a good or excellent school mark (4 or 5 on a scale of 1 to 5) (*N* = 966) (Table 1).

**Table 1:** Sample characteristics according to students’ EFL level.

Level	Boys	Girls	Absent	Total
Elementary	202	184	13	399
Low intermediate	436	517	13	966
Total	638	701	26	1,365

Hungarian students learn FLs as a compulsory school subject from the age of nine. There are some schools which also offer FL courses in earlier school years. Hungarian students can choose from a variety of FLs at school, and most schools offer EFL. Other schools teach German, French, Spanish or Italian as a foreign language. However, the course offerings do not change from school year to school year. They are regulated by the school’s pedagogical documents and curriculum, so parents know in advance what the school offers the students.

### Instruments

In this study, we used the revised version of the Self-Regulated Foreign Language Learning Strategy Questionnaire (SRFLLSQ). The questionnaire was first developed and validated by Habók and Magyar (2018b). A further developed and revised version was used by Habók et al. (2022). It was based on Oxford’s Strategic S^2^R Model and inspired by other measurement tools employed for assessing and validating different dimensions of SRL strategies (e.g., Teng and Zhang, 2018). The questionnaire categories were formed from eight strategy fields: metacognitive (eight items; e.g. “I plan my schedule so I will have enough time to study English”), cognitive (six items; e.g. “I use the English words I know in different ways”), meta-affective (eight items; e.g. “I encourage myself as I learn English so that I can learn what I would like”), affective (eight items; e.g. “It gives me a good feeling when I do well in English”), metasocial (eight items; e.g. “I plan what I want to find out about the cultures of English speakers and/or other cultures through English”), social (six items; e.g. “When I speak with highly proficient speakers of English, I think it is important to get acquainted with their culture”), metamotivational (four items; e.g. “I plan ahead for what I’m going to learn in English in a week or two”) and motivational (four items; e.g. “I use positive self-talk about my reasons for achieving my aims”). The self-report questionnaire employed a five-point Likert scale, ranging from 1 = “Never or almost never true of me” to 5 = “Always or almost always true of me.” The reliability of the questionnaire has been confirmed in previous studies (Habók and Magyar, 2018b). Both CR and CRB ranged from 0.74 to 0.88. The assessment tool was supplemented by background questions inquiring about attitudes to English as well as EFL achievement. As regards the case of attitudes to learning EFL, learners gave their responses on a five-point Likert scale, ranging from 1 to 5. A response of 1 indicated that the student did not like English at all, while a response of 5 meant that the student liked it very much. As regards EFL achievement, they self-reported their school marks from the previous term. The score for EFL achievement can be specified to a five-point scale ranging from 1 = fail, the lowest school mark, to 5 = excellent, the highest school mark.

### Data collection procedure

At the very beginning of the research, we submitted an ethics application to the Institutional Review Board at the University of Szeged Doctoral School of Education. After it was approved, we started the research. There were no ethical issues for the participants; they all agreed and were able to complete the survey. We sent a call with a short description of our research to lower secondary schools, indicating the age group, and opened registration for schools interested in the study. Written informed consent form from parents was handled by the schools before data collection. Once the school registration was completed and the registered classes appeared in our eDia online system, schools received further information on how to access the eDia system, where the data collection was carried out. The schools are familiar with this online system designed, developed and administered by the University of Szeged Centre for Research on Learning and Instruction (Csapó and Molnár, 2019), since our measurements have been carried out on this platform for about 10 years. Students logged in with their official student assessment code to participate in the measurement anonymously. Student names and assessment codes were only seen by the system administrator, who uploaded the data into the system so that students could log in with their identifiers. Students were able to complete the measurement tool during a school lesson in the time frame provided. They needed approx. 15 min to complete the questionnaire. Questionnaire items required students to click on a radio button to complete the measurement tool. Throughout the data collection period, a university system administrator was available to schools in the event of technical problems. In the classroom, teachers supervised the process and aided in solving any technical problems. However, it should be noted that no major technical problems were encountered during the data collection.

### Data analysis

We used IBM SPSS statistics 22.0 to analyze the descriptive statistics. The IBM AMOS 24.0 software package was used to assess CFA and to reinforce the model fit of our hypothesized path model. The construct validity of the measurement model was measured through convergent and discriminant validities. The convergent validity of the scale was tested using average variance extracted (AVE). A value greater than 0.50 provides empirical evidence for convergent validity. The Fornell–Larcker criterion (Fornell and Larcker, 1981) was used to assess discriminant validity. Construct reliability was measured using internal consistency reliability (Cronbach’s alpha) and composite reliability (McDonald’s omega). Values above 0.70 indicate good results, while values above 0.60 are acceptable for empirical research (Hair et al., 2022).

Path analysis was also conducted for these subsamples to ascertain the relationships between EFL strategy use, EFL achievement and attitude to English. The estimations were calculated with the maximum likelihood estimation method. The Chi-square test, comparative fit index (CFI), Tucker–Lewis Index (TLI), normed fit index (NFI) and root mean square error of approximation (RMSEA) were examined to review the goodness-of-fit indices. Values between 0 and 1 for CFI, TLI, NFI and RMSEA were endorsed. If the cut-off value for CFI, TLI and NFI was above 0.90, we accepted the goodness-of-fit indices provided the cut-off value for the RMSEA value was 0.08 or less (Kline, 2015).

## Results

### Validity and reliability analyses

First, confirmatory factor analysis (CFA) was conducted to confirm the construct validity of the questionnaire fields (Chi-square = 12370.533, df = 325, *p* = 0.000, CFI = 0.923, TLI = 0.907, NFI = 0.907, RMSEA = 0.056). The result indicated acceptable fit indices. The eight latent factors were thus established. Construct validity was tested in two parts with the convergent and discriminant validities. Table 2 summarizes the average variance extracted (AVE) values and the correlation coefficients among the factors and for the total questionnaire. All the factors significantly correlated with each other and with the total questionnaire, ranging from 0.32 to 0.68. The lowest coefficient was measured between the metamotivational and cognitive factors (*r* = 0.32), while the highest value was detected between the metasocial and affective factors, on the one hand, and the metacognitive and metamotivational factors, on the other (*r* = 0.68). The moderate correlation coefficients confirm that the factors are distinct from each other. The AVE value is higher than 0.50 in every field, so convergent validity has been confirmed. The square root of AVE is presented on the diagonal line in Table 2, and each of its values is greater than any of its correlations with any other factor. This shows that discriminant validity has also been addressed.

**Table 2:** Average variance extracted (AVE) and correlation coefficients for the SRFLLSQ questionnaire fields.

	AVE	1	2	3	4	5	6	7	8
1. Metacognitive	0.55	0.74							
2. Cognitive	0.60	0.66	0.77						
3. Meta-affective	0.52	0.60	0.54	0.72					
4. Affective	0.51	0.63	0.52	0.49	0.71				
5. Metasocial	0.59	0.59	0.48	0.48	0.68	0.76			
6. Social	0.58	0.55	0.46	0.54	0.51	0.57	0.76		
7. Metamotivational	0.55	0.40	0.32	0.48	0.34	0.40	0.53	0.74	
8. Motivational	0.52	0.68	0.55	0.52	0.64	0.55	0.52	0.37	0.72
9. Total		0.88	0.75	0.78	0.79	0.75	0.76	0.59	0.78

Correlation is significant at the 0.01 level.

Second, the reliability of the questionnaire fields was measured. We found acceptable internal consistency and composite reliability values (Cronbach’s alpha = 0.683–0.885; McDonald’s omega = 0.683–0.885) for each of the strategy fields, which shows that the construct items are acceptable and reliable in measuring the responses. The values for motivational strategies were the lowest, below 0.70; however, in terms of item numbers, they could still be in the acceptable range (Table 3).

**Table 3:** Reliability values for the SRFLLSQ questionnaire fields.

SRL strategy fields	Cronbach’s alpha	McDonald’s omega
Metacognitive	0.813	0.815
Cognitive	0.701	0.710
Meta-affective	0.748	0.754
Affective	0.854	0.852
Metasocial	0.885	0.885
Social	0.850	0.851
Metamotivational	0.742	0.752
Motivational	0.683	0.683

### Descriptive analyses

The mean values for regulation strategy use were examined for the two subsamples. We identified significantly greater mean scores in strategy use in the low intermediate subsample in each strategy field. The students in that subsample reported that they used affective and metacognitive SRL strategies the most. Among the elementary language learners, the motivational and affective strategies showed the greatest use. The strategies used the least in both subsamples were meta-affective strategies (Table 4).

**Table 4:** Descriptive indicators of strategy fields.

SRL strategy fields	M (SD)		*t*	*p* <
	Elementary	Low intermediate		
Metacognitive	2.94 (0.73)	3.61 (0.68)	−16.03	0.001
Cognitive	2.98 (0.71)	3.51 (0.67)	−12.79	0.001
Meta-affective	2.69 (0.75)	3.02 (0.71)	−7.71	0.001
Affective	3.02 (0.84)	3.90 (0.69)	−18.40	0.001
Metasocial	2.65 (0.88)	3.26 (0.89)	−11.42	0.001
Social	2.79 (0.88)	3.42 (0.88)	−11.96	0.001
Metamotivational	2.82 (0.96)	3.20 (0.91)	−6.92	0.001
Motivational	3.03 (0.95)	3.59 (0.83)	−10.13	0.001

We also examined whether there were any significant differences between metastategy and the corresponding SRL strategy use in both subsamples. In the elementary group, the metastrategy use was significantly lower than the regulation strategy use in the following fields: affective (*t* = −11,308, *p* < 0.001), social (*t* = −5,274, *p* < 0.001) and motivational (*t* = −5,198, *p* < 0.001). We found no significant difference between metacognitive and cognitive SRL strategy use. As for the low intermediate subsample, there were significant differences in every field. In the affective, social and motivational fields, metastrategy use was significantly lower (*t* = −44,173, *p* < 0.001; *t* = −10,210, *p* < 0,001; *t* = −14,392, *p* < 0.001; respectively). We revealed significantly higher strategy use of the metacognitive field compared to the cognitive field (*t* = 5,654, *p* < 0.001). Attitude to English in the subsamples was also significantly different. The low intermediate learners preferred English significantly more [M_elem_ = 2.58 (SD = 0.75); M_low intermed_ = 3.32 (SD = 0.66) (*t* = −66,231, *p* < 0.001)].

### Path analyses

We examined the correlations between the questionnaire fields for the subsamples. We confirmed very strong, statistically significant relationships between the strategy fields with their corresponding metafactors. We found significant, strong relations across the questionnaire fields, which also showed significant correlation coefficients with attitude to English and indicated significant relations with proficiency in almost all the cases. We could not identify any significant correlation between motivational strategy use and proficiency among elementary learners, but this was the only case where a significant relation could not be ascertained (Table 5).

**Table 5:** Correlation coefficients for the SRFLLSQ questionnaire fields, attitude and proficiency for the two subsamples.

	1	2	3	4	5	6	7	8	9	10
1. Metacognitive	1	0.75	0.72	0.71	0.65	0.67	0.65	0.53	0.40	0.25
2. Cognitive	0.66	1	0.69	0.67	0.60	0.62	0.57	0.45	0.37	0.20
3. Meta-affective	0.62	0.64	1	0.73	0.68	0.66	0.69	0.67	0.33	0.21
4. Affective	0.66	0.59	0.61	1	0.70	0.70	0.66	0.62	0.48	0.30
5. Metasocial	0.63	0.59	0.64	0.60	1	0.83	0.68	0.59	0.39	0.18
6. Social	0.63	0.58	0.60	0.61	0.86	1	0.71	0.62	0.40	0.20
7. Metamotivational	0.48	0.49	0.64	0.49	0.56	0.59	1	0.64	0.35	0.17
8. Motivational	0.46	0.45	0.58	0.59	0.48	0.53	0.55	1	0.34	0.10
9. Attitude	0.43	0.28	0.22	0.49	0.37	0.37	0.15	0.31	1	0.23
10. Proficiency	0.21	0.18	0.10	0.29	0.13	0.16	n.s.	0.19	0.30	1

Correlation is significant at the 0.01 level: above the elementary subsample and below the low intermediate subsample.

**Figure 2:**
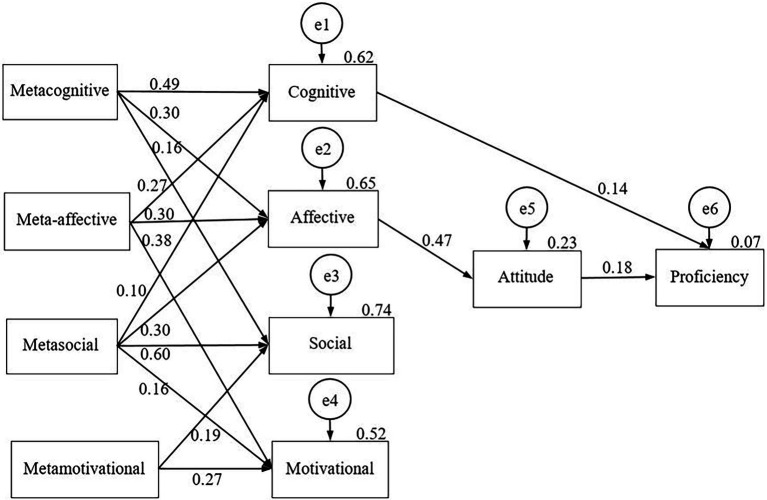
The path model for EFL metastrategies on regulation strategy use, attitude and English proficiency among elementary learners.

**Figure 3:**
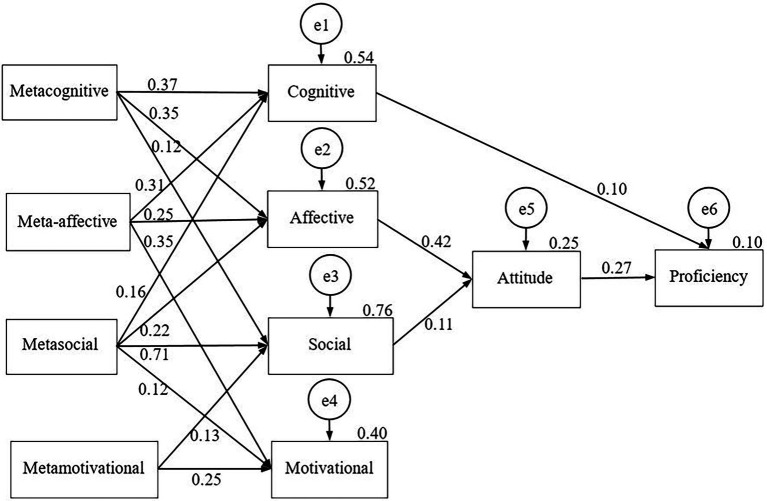
The path model for EFL metastrategies on regulation strategy use, attitude and English proficiency for low intermediate learners.

We examined the effect of the metastrategies on the corresponding SRL strategy fields as well as on EFL achievement through attitudes in our sample. We were looking for the effects of possible paths. We had previously developed a hypothesized model based on Oxford (2017) taxonomy of LLS. The fit indices of the model (Chi-square = 1,628.002, df = 24, *p* = 0.000, CFI = 0.838, TLI = 0.629, NFI = 0.837, RMSEA = 0.220) showed that the hypothesized model did not adequately describe the data. Therefore, we modified our theoretical model and extended the possible paths among the constructs. Furthermore, we expanded the analysis for the two subsamples of the elementary group (Figure 2) and of the low intermediate groups (Figure 3) by constructing a separate model for each group.

For the elementary subgroup, our path model this time indicated acceptable fit indices (Chi-square = 46.009, df = 18, *p* = 0.000, CFI = 0.990, TLI = 0.968, NFI = 0.983, RMSEA = 0.063). Therefore, we concluded that the metastrategies significantly influenced other non-meta SRL strategy types. The metasocial strategies had the most powerful effect, with a significant direct effect on all other non-meta fields. The affective strategies followed a direct path to attitude to English. Attitude to English determined EFL achievement directly. A direct path from cognitive strategies was also observed on EFL achievement. The metacognitive, meta-affective and metasocial fields generated mediating effects on proficiency through the cognitive strategies.

As for elementary learners, we also investigated the explained variance of the metafactors on strategy use. We found that three strategy fields explained the cognitive strategies: the metacognitive, meta-affective and metasocial fields. In total, the explained variance was 62%. These three metafactors also explained 65% of the affective strategies. As for the social field, the metacognitive, metasocial and metamotivational fields explained 74% of it, while the meta-affective, metasocial and metamotivational fields explained 52% of the motivational strategies.

For language attitude as a dependent variable, we accounted for the considerable direct effect of affective strategies and the indirect effects of the metacognitive, meta-affective and metasocial fields. The observed effects represented 0.23% of the total effects. A much lower effect of strategies and FL attitude was found on proficiency. Strategies and attitudes only explained 7% of students’ FL proficiency.

As for the low intermediate subsample, we found similar results; however, the RMSEA value fell to slightly above the cut-off value (Chi-square = 146.307, df = 17, *p* = 0.000, CFI = 0.997, TLI = 0.926, NFI = 0.974, RMSEA = 0.089). Several direct effects of metastrategies were observed on the SRL strategies, the same structure as that for the path model for the elementary group (Figure 2). Attitude to English was also directly influenced by the affective and social strategies, and it had a significant direct impact on English proficiency. A direct path for the cognitive strategies was also observed; the cognitive SRL strategies also explained English proficiency.

As for the low intermediate learners, we also discovered significant effects of metacognitive, meta-affective and metasocial factors on cognitive strategies. The total known impact was 54%. An effect of metacognitive, meta-affective and metasocial factors was also identified on affective strategy use. These metafactors explained 52% of the total effects. The effects on the social strategies were found to be the greatest. On the whole, the metacognitive, metasocial and metamotivational effects explained 76% of all known effects. As for the motivational factor, the meta-affective, metasocial, and metamotivational fields explained 40% of it.

We explained a smaller proportion of effects on attitudes than on social strategies. Among our independent variables, affective and social strategy use explained 25% of the total effects on attitude. Ten per cent of proficiency was described through attitude to English and other strategy factors. A significant direct path from the cognitive strategies was identified on proficiency. Proficiency was also indirectly influenced by metacognitive, meta-affective and metasocial strategies mediated by the cognitive field.

## Discussion

The study aimed to explore the structural relationships of self-regulated LLS and proficiency through attitude to English among students of elementary and low intermediate levels of proficiency based on the self-regulated language learning theory. Specifically, we were looking for the answer to how metastrategy use affects the corresponding self-regulated LLS and how strong this effect is on the students’ proficiency level.

In our research, we employed the revised version of the Self-Regulated Foreign Language Learning Strategy Questionnaire (SRFLLSQ), developed and validated by Habók and Magyar (2018b) in previous work. The development of the questionnaire constructs was inspired by Oxford’s Strategic S^2^R Model (2017). The revised version was administered by Habók et al. (2022). In our study, we concluded that the internal consistency, composite reliabilities and construct validity of all the questionnaire fields were acceptable. It can be concluded that a new questionnaire on self-regulated language learning strategies has therefore been developed, which is suitable for use with upper secondary school students. In the field of writing, for example, Teng and Zhang (2016) validated a new measurement tool for strategy use called the Writing Strategies for Self-Regulated Learning Questionnaire.

Our first research question focused on the extent of regulated LLS use in the two subsamples. The results showed that in each strategy field the low intermediate group employed the SRL strategies more. According to their report, the affective and metacognitive SRL strategies were employed the most. Among the elementary language learners, the motivational and affective strategies were used the most. This may be due to the fact that beginners in language learning rely more on their feelings (such as relaxing or encouraging themselves and enhancing self-confidence) and that motivation (rewarding themselves for good progress, positive self-talk or positive self-belief) is a stronger factor in their achievement. Learners at higher levels more strongly rely on their metacognitive strategies, such as planning, organizing and monitoring their cognitive processes. The use of meta-affective strategies was the least characteristic of both subsamples, thus implying that students at this age are less concerned with such strategies.

A comparison with Bessai (2018) findings partly reinforces the results of our research. In her study, she found that less proficient students reported a greater use of compensation strategies, a result which was not borne out by our study. However, she also pointed to frequent use of metacognitive strategies among more proficient learners, a result which is consistent with our findings. Lee and Heinz (2016) also demonstrated that metacognitive strategies were used more in the more proficient group. Chen and Aziz (2021) likewise confirmed a metacognitive strategy preference among students with high academic achievement. Further, Sánchez (2019) discovered the highest mean values in the fields of social and metacognitive strategies for both low and high achievers, a finding which is also partly consistent with our results. Our findings confirm the complex nature of SRL and focus our attention on the metacognitive, affective and motivational components of EFL learning (Winne and Hadwin, 1998, 2008; Perry and Winne, 2006; Efklides, 2011; Panadero, 2017).

Our second research question concerned attitude to the English language in the subsamples. The central role of attitude has been reinforced in previous research (Platsidou and Kantaridou, 2014; Habók and Magyar, 2018b; Getie, 2020; Habók et al., 2022). We discovered significantly different attitudes in the subgroups. Specifically, the low intermediate learners had a greater preference for English, a finding which has partly been confirmed by some studies (Habók and Magyar, 2018b; Habók et al., 2022).

To answer the third and fourth research questions, we developed two theoretical models for the separate groups of students with different proficiency levels. The two models were identical in the sense that the same constructs were employed and almost the same direct and indirect effects were justified. The only difference was the direct effect of the social field on attitude in one case, which could not be confirmed in the elementary sample. Otherwise, the level of impacts was also similar.

As to the question of how the use of metastrategies affects the corresponding self-regulated LLSs, we found that it had a significant effect on the corresponding factors in all of the metastrategy fields. The same direct effects were found in both models, and there were no significant differences in the standardized coefficients. The metasocial field significantly influenced all of the regulated strategy types in both models. The metacognitive and meta-affective fields influenced three types of SRL strategies, and the metamotivational field had direct effects on the motivational and social strategies. On the whole, the positive contribution of the metastrategies on regulated strategy fields appeared to be robust, thus underlining recent LLS research that emphasizes the role of metafactors in the language learning process (Habók and Magyar, 2018a; Habók et al., 2022).

The last research question addressed the effect of SRL on students’ proficiency level. According to the model indicators, there were differences between the direct effects of the models for the more and less proficient groups. As for the model for the elementary subsample, social strategies had no significant direct effect on students’ attitude. This may be due to the fact that social strategies, such as starting a conversation in English or insufficient knowledge of the other culture, lead elementary learners to feel discouraged, causing them to use these strategies in a restricted manner.

In both models, a significant direct influence of cognitive strategies was identified on proficiency. Proficiency was also indirectly influenced by the metacognitive, meta-affective and metasocial strategy fields, mediated by the cognitive field. In total, strategies and attitudes explained 7–10% of students’ FL proficiency. These findings are consistent with a number of relevant studies (Ardasheva, 2016; Teng and Zhang, 2018; Taheri et al., 2019). Ardasheva also found that among the six LLS categories she has investigated, only the metacognitive strategy field had a significant direct effect on English proficiency (Ardasheva, 2016).

One of the interesting findings is that the motivational factor turned out to be isolated in the sense that although it was directly affected by the corresponding metamotivational and two other factors of strategies, it had no further effect either on attitude or proficiency. The reason may be that motivational strategy constitutes a distinct factor and the role of the strategies involved is somewhat different in predicting language proficiency. The findings correspond to similar previous research (Ardasheva, 2016; Teng and Zhang, 2018; Habók and Magyar, 2018a), which also emphasized the significance of motivation in self-regulated processes (Schunk and Zimmerman, 2008; Boekaerts, 2011; Schunk and Greene, 2018).

## Conclusion

In summary, our research contributes to and strongly supports the significance of strategy research through self-regulatory perspectives, thus expanding our understanding of FL learning. The study has reinforced the notion that FL learners can become more successful and effective in their learning by employing certain self-regulated LLSs. Our work also highlights the fact that students’ ability to regulate their learning process along with the use of various learning strategies has a definite influence on their proficiency. The research design with separate models confirmed that both elementary and low intermediate learners can benefit from self-regulated strategy use, although at different levels and with different effects. The study also pointed out the mediating effect of attitude to English in the learning process, which can directly influence proficiency in that language. It can be concluded that the outputs of the research may represent a valuable contribution to classroom research and for language teachers.

## Pedagogical implications

Our research also has critical implications for teaching practice. At the lower secondary level, it is very important to recognize that learning is also determined by metafactors. What a learner thinks about a learning activity, how much he or she likes a FL and how well he or she performs in it also determine strategy use. Teachers are thus encouraged to assign a variety of tasks to students to pave the way for them to make observations while completing a task. Teaching new self-regulatory techniques and strategies can improve students’ self-regulatory learning skills and capacities. Several approaches can be used, either embedded in the language classroom or outside compulsory education as a form of out-of-school activity. Teachers must encourage students to employ more metacognitive strategies, to self-evaluate their progress or to self-assess their performance. Adaptive learning methods are recommended that are tailored to different student levels. Another important area is to exploit the potential of certain motivational factors that also need to be embedded in classroom practices. As a result, students become autonomous learners, can set their own aims and proactively regulate their language learning process, thus becoming lifelong learners.

## Limitations

Despite the significance of the research, some limitations have been identified. First, our data was collected among Year 8 students, so we cannot generalize it to all grades and language learning proficiency levels. A longitudinal or cross-sectional study would be needed to provide a comprehensive overview of self-regulated LLS use. Second, we could not fully optimize the RMSEA index of our model for the low intermediate subsample. Third, in the future, by employing additional factors that influence language learning (self-efficacy, self-concept, etc.), we will be able to arrive at a more nuanced description of the role of diverse factors in determining learning outcomes. Fourth, motivational regulation strategies formed a separate factor without any direct effect on either attitude or proficiency. We assume that this can only be due to the segment of the motivational construct under investigation. Further examination with an extended view of motivational factors (intrinsic, extrinsic, mastery self-talk, interest enhancement, etc.) is called for. Fifth, qualitative or mixed-method research designs involving observations or behavioral measures would be recommended to gain an in-depth insight into how learners think while completing proficiency-based tasks and to analyze the degree to which these processes are in line with learners’ responses to questionnaire items.

## Data availability statement

The original contributions presented in the study are included in the article/supplementary materials, further inquiries can be directed to the corresponding author.

## Ethics statement

The studies involving human participants were reviewed and approved by Institutional Review Board at the University of Szeged Doctoral School of Education. Written informed consent to participate in this study was provided by the participants’ legal guardian/next of kin.

## Author contributions

AH and AM designed the study, implemented the data collection, performed the statistical analysis, and managed the process of writing the manuscript. GM supervised the research and provided support. All authors contributed to the manuscript revision and approved the final version of the manuscript.

## Funding

This research was supported by a Hungarian National Research, Development, and Innovation Fund grant (under the OTKA K135727 funding scheme) and a Hungarian Academy of Sciences Research Program for Public Education Development grant (KOZOKT2021-16).

## Conflict of interest

The authors declare that the research was conducted in the absence of any commercial or financial relationships that could be construed as a potential conflict of interest.

## Publisher’s note

All claims expressed in this article are solely those of the authors and do not necessarily represent those of their affiliated organizations, or those of the publisher, the editors and the reviewers. Any product that may be evaluated in this article, or claim that may be made by its manufacturer, is not guaranteed or endorsed by the publisher.
